# Occurrence of overt seizures in comatose survivor patients treated with targeted temperature

**DOI:** 10.1002/brb3.842

**Published:** 2017-10-18

**Authors:** Anda Eilam, Volodymyr Samogalskyi, Gennady Bregman, Sarit Eliner‐Avishai, Ronit Gilad

**Affiliations:** ^1^ Neurology Department Kaplan Medical Center Rechovot Israel; ^2^ Intensive Care Unit Kaplan Medical Center Rechovot Israel; ^3^ Medical Management Kaplan Medical Center Rechovot Israel; ^4^ The Hebrew University Jerusalem Israel

**Keywords:** hypothermia, mortality, postanoxic seizures

## Abstract

**Background:**

Unconscious patients after out‐of‐hospital cardiac arrest have a high risk of death. Therapeutic hypothermia is recommended by international resuscitation guidelines in order to attenuate secondary destructive physiological processes such as reperfusion injury, apoptosis, and cerebral edema. The target temperature to reach ranges between 32 and 34°C for at least 24 hr. Hypothermia can induce metabolic disturbances. There are some reports in the literature indicating the presence of seizures during targeted temperature management. On the other hand, postanoxic seizures are a sign of unfavorable neurological outcome. The purpose of this study was to evaluate the occurrence of overt seizures in comatose survivor patients treated with targeted temperature in respect to overt seizures in a normal temperature group of comatose patients.

**Methods:**

This was a retrospective study of unconscious adults post cardiopulmonary resuscitation, hospitalized in the intensive care unit during the years 2008–2015. The patients were divided into two groups: those treated with hypothermia and those with normal body temperature. Both groups were evaluated for the appearance of overt seizures during their hospitalization which was the primary outcome of the study.

**Results:**

The data of 88 consecutive unconscious patients after out‐of‐hospital cardiac arrest were collected. Twenty‐six patients were treated with targeted temperature (32–34°C) and 62 patients with normal temperature. In the hypothermic group, 6 (23%) patients developed overt seizures during hospitalization compared to 11 (17%) in the normothermic group. The mortality rate was similar in both groups, 16 (61%) in the hypothermic group and 38 (61%) in the conservative group. According to the present study, overt seizures were more common in the group treated with hypothermia.

## 1 INTRODUCTION

The annual incidence of out‐of‐hospital cardiac arrest in industrialized countries has been estimated to be 92–189 cases per 100,000 inhabitants (Neumar et al., 2008). Perhaps the most important manifestations of the postcardiac arrest syndrome are neurologic (Bassetti et al., 1996). The chance of neurologically intact survival from cardiac arrest remains low despite significant advances in clinical care, including cardiopulmonary resuscitation (CPR) and treatment with therapeutic hypothermia. Less than half of those with return to spontaneous circulation survive to hospital discharge and 20%–30% of the survivors are neurologically devastated (Bisschops et al., 2011; Wijdicks et al., 2006). Seizures have been reported in 10%–12% of the survivors and have been shown to be an independent risk factor for mortality (Arnoldus & Lammers, 1995; Celesia, Grigg, & Ross, 1988; Fugate et al., 2010; Harper & Wilkes, 1991; Hui et al., 2005; Kawai, Thapalia, & Verma, 2011; Krumholz, Stern, & Weiss, 1988; Mani et al., 2012; Rabinstein & Wijdicks, 2012; Rossetti et al., 2007; Wijdicks, Parisi, & Sharbrough, 1994; Young, Jordan, & Doig, 1996).

Therapeutic hypothermia is recommended by international guidelines, but the supporting evidence is limited (Bisschops et al., 2011; The Hypothermia After Cardiac Arrest [HACA] study group, 2002; Polderman et al., 2003; Silfvast et al., 2003). Hypothermia has antiepileptic effects, and yet seizures in patients recovering from cardiac arrest can occur despite therapeutic hypothermia, and furthermore, the risk of seizures may increase during rewarming. Any information on seizure incidence in comatose patients treated with targeted hypothermia is scarce (Karkar et al., 2002; Maeda, Hashizume, & Tanaka, 1999; Rittenberger et al., 2012; Rossetti et al., 2007; Synek, 1990).

The goal of the present study was to assess the incidence of overt seizures in comatose survivors of cardiac arrest treated with therapeutic hypothermia and to characterize the types of seizures encountered and their impact on the patients’ outcome.

## 2 MATERIAL AND METHODS

### 2.1 Study design, setting, and selection of participants

**Figure 1:**
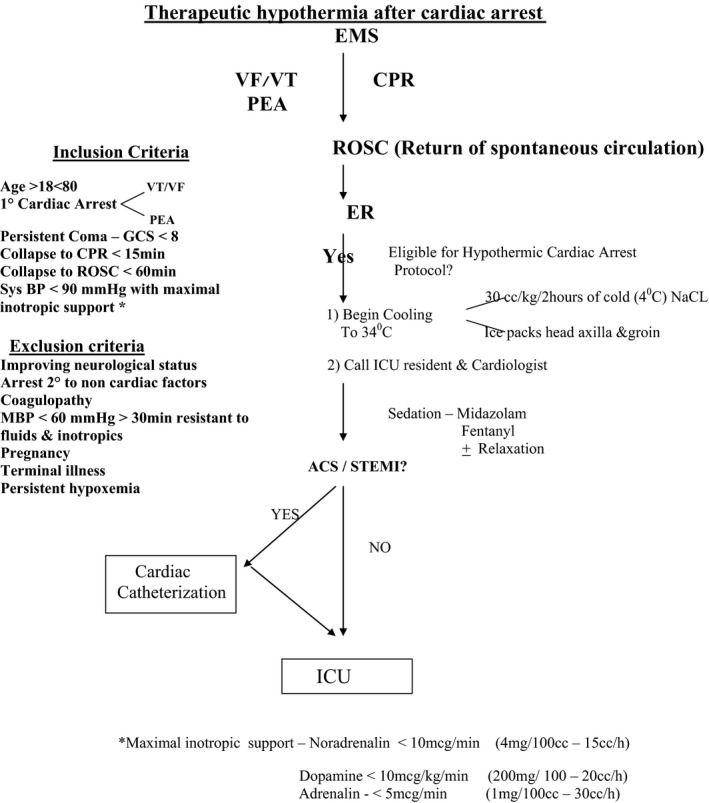
Therapeutic hypothermia after cardiac arrest

The study was conducted at an urban, academic medical center with approximately 75,000 annual adult visits in the emergency department. The selection of patients suitable for therapeutic hypothermia and the protocol for application of therapeutic hypothermia were at the discretion of ICU team in our center. All of the patients treated with therapeutic hypothermia and part of the other cardiac arrest after CPR survivors were initially admitted and treated during the first days of hospitalization in the ICU. The flowchart was the protocol used for therapeutic hypothermia in our institution (Figure 1).

After approval by the institutional review board, a retrospective chart review of every cardiac arrest patient (18 years or older), who was admitted to the ICU in our center between 1 January 2008 and 31 December 2015, was conducted.

### 2.2 Data collection

Patients for the study were identified from the “chameleon” database. (The chameleon database contains data of all the hospitalizations in the Kaplan Medical Center in the last 10 years.) The search contained the following terms: cardiac arrest, ventricular tachycardia, ventricular fibrillation plus CPR, which was performed.

Participants’ charts were initially screened to ensure patients were admitted to the ICU following CPR after cardiac arrest, and the patients were older than 18 years of age.

Patients with previous history of seizures or experiencing seizures immediately after the CPR were excluded from the series. Overt seizures that were collected included tonic‐clonic and myoclonic seizures. The authors reviewed description of seizures for each patient. At least one of the authors was involved in the treatment, and follow‐up of the patients described and observed the seizures.

Data were collected regarding demographic information—age, gender, comorbidities, and causes of cardiac arrest. Additional data collected included brain CT results and concomitant medications. Patients were divided into two groups: those treated with hypothermia and those with normal body temperature. Both groups were evaluated for the appearance of overt seizures during hospitalization in the ICU, which was the primary outcome of the study. Seizures were characterized as generalized tonic‐clonic, myoclonic, or partial seizures.

EEG recordings were reviewed in those cases having been performed. EEG recordings were performed only in those patients with overt seizures. Secondary outcome end point was mortality during hospitalization.

### 2.3 Data management

Study data were collected on paper case report forms. It was analyzed using JMP 12.0.1 (SAS Institute Inc. 2015). The groups were compared regarding gender distribution by the Pearson's chi‐square test, and regarding age, by *t* test. Nominal logistic regression was used to compare the groups regarding seizures and mortality, after correcting for age and gender differences.

## 3 RESULTS

Between 2008 and 2015, 88 consecutive patients, who arrived at the ER after cardiac arrest and CPR, were admitted to the ICU at the Kaplan Medical Center. The mean age was 56.3 years (range 19–86). Of these, 47 (53%) were males. Performance of hypothermia or not was at the discretion of the ICU team as there were no guidelines at that time (Tables 1, 2, 3).

**Table 1:** Characteristics of the cohort

Total no.	88
Mean age	56.3 years
Sex	41F, 47M
Median Glasgow coma score	6
Diagnosis	
VF	11 (12.5%)
Asystole	40 (45%)
Acute myocardial infarction	14 (16%)
Asthma	5 (6%)
Others	18 (20%)
Brain CT	
Brain edema	19 (22%)
Intracerebral hemorrhage	2 (2%)
No findings	67 (76%)
EEG	
SE	3
Slowing	9
Low amplitude	4

**Table 2:** Patients’ characteristics by group

	Normothermia	Hypothermia
Age	61 (21–86)	51 (19–68)
Gender	29 women	7 women
Median Glasgow coma score	6	7
	47%	27%

**Table 3:** Outcome by group

Outcome	Normothermia	Hypothermia
Seizures	10	6
	16%	23%
Death	37	16
	60%	61%

Characteristics of patients in the two groups (hypothermic and normothermic) are shown in Table 2. The mean age in the hypothermic group was significantly lower than in the normothermic group (*p* = .0149). The differences between gender distribution were borderline significant (*p* = .0546).

The condition that caused cardiac arrest was obvious in 80%. Most of the patients experienced cardiac conditions which were evident in the recorded primary ECG strip; asystole being the most common. Five patients experienced cardiac arrest secondary to asthma attack. In 20% of the patients, there were several other causes like massive PE, tamponade, or the condition causing cardiac arrest was unknown.

Brain CT was performed during the initial work‐up in all patients. Most of the scans (76%) were interpreted as normal. Brain edema was in seen 22% of the patients. In two patients, intracerebral hemorrhage was present.

EEG was performed only in patients with seizures, being performed between several minutes and until several hours after the initial observed seizure. Most of the studies showed nonspecific slowing of the background or low amplitude findings, reflecting most probably the brain dysfunction (postanoxic encephalopathy). In several patients interictal epileptiform activity was seen. In four patients rhythmic PEDS were seen.

The following outcomes are shown in Table 3: the percentage of patients who died during the hospitalization was 60% and the percentage of survivors was similar in the hypothermic (39%) and the normothermic group (40%) (*p* = .52), corrected for age and gender differences; 6 (23%) patients in the hypothermic group experienced seizures versus 10 (16%) in the normothermic group (*p* = .70), corrected for age and gender differences.

As expected from previous studies, mortality in the group with seizures was higher compared to the group without seizures (69% vs. 46%). This difference was distinctive in the group that was treated with hypothermia (83% mortality in the group with seizures vs. 55% in the group without).

There were no differences between the groups in terms of ROSC or clinical examination at hospital arrival.

## 4 DISCUSSION

These data suggest that overt seizures occur in 18% of the patients admitted to ICU after CPR for cardiac arrest. This percent is slightly lower than the usually 30% reported in literature and may represent the fact that only clinically overt seizures were reported in our study, as opposed to studies which used continuous EEG monitoring. On the other hand, the impact of electrographic seizures on the mortality has yet to be determined, since most of the studies reported a negative impact of overt seizures on patients’ outcome.

The percent of patients seizing in the hypothermic group was 23%, which was higher than that in the normothermic group. This difference did not reach statistical significance due to the small number of patients with seizures in both groups, but is interesting because of the alleged neuroprotective effect attributed to hypothermia. This is more so when keeping in mind that all the patients in the hypothermic group were treated with benzodiazepines during the induction of hypothermia, in contrast to the normothermic patients. Also, the hypothermic group tended to include younger patients with expected better prognosis, which makes the increased seizure incidence more unexpected in this group. As patients were continuously monitored for electrolyte disturbances (such as decreases in the levels of K, Ca, Mg, or P), this cannot be counted as responsible for the increased seizure rate.

This study has several limitations: it is a retrospective one and included a small number of patients in each group, not enough to reach statistical significance for the primary or secondary end points. The study groups were formed by the selection of the ICU staff and were not equivalent in terms of demographic data or causes of cardiac arrest. In addition, the patients were not continuously monitored with EEG, so that electrographic seizures may have been missed.

## 5 CONCLUSIONS

In this study, overt seizures occurred in 23% of the patients, who underwent hypothermia after cardiac arrest, and this percent was higher than the 16% in the normothermic group. As reported in previous studies, seizures in postanoxic patients were associated with increased mortality.

Keeping the limitations of the study in mind, the outcome of the hypothermic group in terms of seizures is still surprising. As most of the seizures appeared during or immediately after rewarming, a possible explanation may be a rapid increase in brain metabolism in this period. Hypothermia can be a two‐edged sword: although significant benefits can be achieved, there are many potential side effects that, if left untreated, can diminish or even negate the potential benefits. Further double blind, prospective studies are needed to evaluate the impact of hypothermia on the incidence of postanoxic seizures.
